# Identical Assemblage of *Giardia duodenalis* in Humans, Animals and Vegetables in an Urban Area in Southern Brazil Indicates a Relationship among Them

**DOI:** 10.1371/journal.pone.0118065

**Published:** 2015-03-11

**Authors:** Cristiane Maria Colli, Renata Coltro Bezagio, Letícia Nishi, Thaís Souto Bignotto, Érika Cristina Ferreira, Ana Lúcia Falavigna-Guilherme, Mônica Lúcia Gomes

**Affiliations:** 1 Environmental and Food Parasitology Laboratory, State University of Maringá (UEM), Paraná, Brazil; 2 Chemical Engineering Department, UEM, Paraná, Brazil; 3 Engineering and Exact Sciences Centre, State University of West Paraná, Paraná, Brazil; 4 Statistics Department, UEM, Paraná, Brazil; University of Brighton, UNITED KINGDOM

## Abstract

**Background:**

*Giardia duodenalis* infects humans and other mammals by ingestion of cysts in contaminated water or food, or directly in environments with poor hygiene. Eight assemblages, designated A–H, are described for this species.

**Methodology/Principal Findings:**

We investigated by microscopy or by direct immunofluorescence technique the occurrence of *G*. *duodenalis* in 380 humans, 34 animals, 44 samples of water and 11 of vegetables. *G*. *duodenalis* cysts present in samples were genotyped through PCR-RFLP of β giardin and glutamate dehydrogenase (*gdh*) genes and sequencing of *gdh*. The *gdh* gene was amplified in 76.5% (26/34) of the human faeces samples with positive microscopy and in 2.9% (1/34) of negative samples. In 70.4% (19/27) of the positive samples were found BIV assemblage. In two samples from dogs with positive microscopy and one negative sample, assemblages BIV, C, and D were found. Cysts of *Giardia* were not detected in water samples, but three samples used for vegetable irrigation showed total coliforms above the allowed limit, and *Escherichia coli* was observed in one sample. *G*. *duodenalis* BIV was detected in two samples of *Lactuca sativa* irrigated with this sample of water. BIV was a common genotype, with 100% similarity, between different sources or hosts (humans, animals and vegetables), and the one most often found in humans.

**Conclusions/Significance:**

This is the first study in Brazil that reports the connection among humans, dogs and vegetables in the transmission dynamics of *G*. *duodenalis* in the same geographic area finding identical assemblage. BIV assemblage was the most frequently observed among these different links in the epidemiological chain.

## Introduction

*Giardia duodenalis* (Syn. *G*. *lamblia* and *G*. *intestinalis*) is the flagellate protozoan responsible for giardiasis, a disease that is found in the general population, particularly in school-age children, worldwide [1,2]. Giardiasis has a public health impact because of its high prevalence and propensity to cause major outbreaks and emergency responses, in addition to its effects on the growth and cognitive functions of infected children [2].

The cysts, resistant forms, contaminate water or food, or the transmission occur through direct person-to-person contact in environments with compromised hygiene levels, such as childcare centres [3,4]. Water is an important vehicle if ingested directly or used in the irrigation and preparation of foods consumed raw [5,6].

In an epidemiological context it is important to detect the presence of the parasite, the genotype circulating in a given area and the relationship among the different links of the chain of transmission. Currently, it is accepted that there are eight genotypes or assemblages of *G*. *duodenalis* that are morphologically identical but genetically distinct (A–H) [2,3,7,8]. Assemblages A and B generally infect humans, but can infect other vertebrates, and therefore are considered to be zoonotic. Assemblages C–H are more host-specific: C and D in dogs, E in hoofed livestock, F in cats, G in rats and H in seals [7–9].

Little is known about the geographic distribution of the zoonotic genotypes (A and B) between different geographical areas in Brazil [10–13]. An important aspect of the epidemiology of giardiasis is to understand the potential of cross-transmission among hosts that harbour different genotypes of *Giardia*, the risk and environmental factors involved in exposure to this pathogen and its zoonotic potential.

In this context, we investigated the occurrence of *Giardia duodenalis* and their genotypes in humans, animals, water and vegetables, to check the relationship among them in the epidemiological chain of an urban area of Southern Brazil.

## Material and Methods

### Study area

The study was conducted from March 2012 to June 2013 in the municipality of Ângulo (23° 11’ 41”S; 51° 54’ 55”W), situated in Paraná State, Southern Brazil. This municipality presents a population of 2,859 inhabitants, a degree of urbanization of 78.8% and a score on the human developmental index of 0.721 [14].

### Ethics statement, population and variables studied

This study was approved by the State University of Maringá Ethics Committee of Research Involving Human Beings (Comitê de Ética em Pesquisa Envolvendo Seres Humanos—COPEP/UEM-439/2009) and the Ethics Committee on Animal Use in Experimentation (Comitê de Conduta Ética no Uso de Animais em Experimentação—CEAE/UEM-130/2009). Each individual, or their guardian, signed the consent form approved by COPEP, agreeing to participate in the study. In total, 380 individuals of both genders, with ages ranging from 5 months to 78 years, were investigated. The study population comprised children and adolescents from the three schools (municipal, state and day care) operating in the municipality. A random sample of students was calculated considering the number of students enrolled in each school, a 95% confidence level, 5% error, and 29% prevalence for giardiasis [15]. The remaining samples consisted of adults, including school staff, families of children with positive results for *G*. *duodenalis*, vegetable producers, senior citizens, and other city residents who learned of the study and wanted to participate voluntarily. Some of these participants who had animals in house also provided faecal samples of their pets for analysis. All those infected were referred for aetiological treatment by physicians through the management of Health Surveillance/Municipal Secretariat of Health of Ângulo.

The data regarding variables, age, gender, presence, number and species of pets in the household, type of water consumed, habit of consuming vegetables, were obtained through a structured questionnaire.

### Samples origin and isolation of *Giardia* spp cysts

**Detection and isolation of** ***G*. *duodenalis*** **cysts in faeces**. Faecal samples from humans (n = 380) and domestic animals (32 dogs and 2 cats) were collected only once. All participants received a polypropylene flask with screw cap, cleaned and labelled and without preservatives, and the instructions on how to collect and identify the faecal material. The samples were processed by spontaneous sedimentation in water. After 24h, 3 mL of faecal pellets were analysed by the Ritchie method [16], modified by replacing formaldehyde with distilled water. The sediment obtained was examined by optical microscopy. DNA was extracted from all animal samples with positive or negative results on microscopy, from all positive human samples (34) and from an equal number of randomly selected negative human samples (34). The β-giardin (*bg*) and glutamate dehydrogenase (*gdh*) genes were amplified from these samples and the *gdh* gene was sequenced as described below.

**Water samples and immunologic, molecular and microbiologic analyses.** Samples of fifty litters of water used for vegetable irrigation or human consumption were collected only once, into flasks sanitized with 10% sodium hypochlorite solution, at each of the 44 collection points. The water used for human consumption came from two artesian wells existing in the municipality and were treated with only chlorination before water distribution. Samples A1, A2 and A3 were used for the irrigation of vegetables. The samples A4 and A5 were collected before treatment from the output of each of the wells and A6 was collected in the output from reservoir after treatment. Samples A7, A8 and A9 were collected from water troughs in the three schools and A10 to A44 were collected from water taps in the residences located in different parts of the municipality, including all those (35) where residents presented positive results for *G*. *duodenalis*.

For immunologic analyses the water samples were processed by means of a membrane-filtration technique, with mechanical extraction and elution followed by centrifugation [17,18]. From the sediment resuspended in 500 μL of distilled water, 5 μL was used for the direct immunofluorescence technique and a confirmatory test with the inclusion of the fluorogenic vital stain DAPI (4’, 6’-diamidine-2-phenylindole; Sigma Chemicals Co., St Louis, MO, USA), using the Merifluor commercial kit (Meridian Bioscience, Cincinnati, OH, USA) [17,18].

For all samples the DNA was extracted from the sediment (100 μL) and used in the amplifications by polymerase chain reaction (PCR) and restriction fragment length polymorphism (PCR-RFLP) of the *bg* and *gdh* genes and sequencing of the *gdh* gene, as described below.

Based on the findings of Nishi et al (2009) [18] and Karon et al (2011) [19] physical-chemical and microbiologic analyses were proposed, evaluating colour, turbidity, pH and contamination by *Escherichia coli* and total coliforms in accordance with the Standard Methods for the Examination for Water and Wastewater [20]. The parameters for the quality of water for human consumption were established by Ordinance 2914/2011 of the Ministry of Health [21] and parameters for irrigation were verified according to Resolution No. 357 of the National Environmental Council [22], which establishes in Article 4 that water used for the irrigation of vegetables consumed raw is classified as freshwater class 1.

**Detection and isolation of** ***Giardia*** **spp in vegetables.** Six samples of lettuce (*Lactuca sativa*) and five of wild chicory (*Cichorium intybus intybus*) produced from a single area in the municipality were collected only once, on the same occasion on which the samples of water used for vegetable irrigation were collected. The samples were collected in new plastic bags, transported in thermal boxes and analysed at the Environmental and Food Parasitology Laboratory of the State University of Maringá (LPAA/UEM). From each vegetable, a 50 g sample, preferably obtained from the outer leaves, was washed with 100 mL of 1% Tween 80 by hand shaking for one minute. The resulting liquid was processed in the same way as the water samples.

### Molecular analyses

**Extraction of DNA and amplification of the β-giardin and glutamate dehydrogenase genes.** The PureLink PCR Purification kit (Invitrogen, Carlsbad, CA, USA) was used to extract DNA from the cysts according to the recommendations of the manufacturer [23]. A fragment of 753 bp (base pairs) from the *bg* gene was amplified with the primers G7 (5′AAGCCCGACGACCTCACCCGCAGTGC3′) and G759 (5′GAGGCCGCCCTGGATCTTCGAGACGAC-3′) [24] with modifications as previously described [23]. To obtain a fragment of approximately 432 base pairs from the *gdh* gene, a semi-nested PCR was performed using the following primers: external forward primer GDHeF (5’TCAACGTYAAYCGYGGYTTCCGT3’), internal forward primer GDHiF (5’CAGTACAACTCYGCTCTCGG3’), and reverse primer GDHiR (5’GTTRTCCTTGCACATCTCC3’) [25] with modifications. Each amplification reaction was performed in a final volume of 11 μL, containing buffer 10× (200 mmol/L Tris-HCl pH 8.4, 500 mmol/L KCl, 1.5 mmol/L MgCl2, 1.5 U of Platinum *Taq* DNA Polymerase (Invitrogen) for the *bg* marker and 0.5 U for the *gdh* marker, 200 μmol/L of triphosphate deoxyribonucleotides, 2 pmol of each primer, sterile Milli-Q H2O, and 2 μL of total DNA. The conditions for amplification of the *bg* marker were: denaturation at 94°C for 5 min, followed by 35 cycles at 94°C for 30 s, 65°C for 30 s, 72°C for 60 s and a final extension at 72°C for 7 min, and for the *gdh* marker were: denaturation at 94°C for 2 min, followed by 35 cycles at 94°C for 45 s, 55°C for 30 s, 72°C for 45 s and a final extension at 72°C for 5 min, in the first and second reactions. The amplified products were visualized in 5.0% polyacrylamide gels, silver stained, and digitally recorded.

**Genotyping and sequencing.** *Giardia* genotyping was carried out using PCR-RFLP for both the *bg* and *gdh* genes, and DNA sequencing for the *gdh* gene. For PCR-RFLP assays, amplified products of *bg* and *gdh* genes were digested, respectively, with five units of the *Hae* III restriction enzyme (New England Biolabs Inc., USA) for 4 h at 37°C [23] and two units of *Nla* IV endonuclease (New England Biolabs Inc., USA), for 3h at 37°C [25]. Restriction fragments were visualized in 5.0% polyacrylamide gels, silver stained, and digitally recorded.

For sequence analysis, semi-nested PCR products (*gdh* gene) were purified using the PureLink PCR Purification Kit (Invitrogen, Carlsbad, CA, USA) according to the manufacturer’s instructions. The amplicons (*gdh* gene) were sequenced in both directions according to the manufacturer’s instructions with the BigDye Direct Cycle Sequencing Kit (Life Technologies, Carlsbad, CA, USA) in an automated DNA sequencer—3500xL Genetic Analyzer (Applied Biosystems).

**Sequence analysis.** Nucleotide sequences, shaped as a chromatogram, were aligned using Clustal W [26], edited with BioEdit Sequence Alignment Editor 7.0.1 [27] and, when necessary, adjusted manually. BLAST software (http://www.ncbi.nlm.nih.gov/blast/) was used for comparison of the nucleotide sequences analysed in the present work with sequences available at GenBank. The sequences of the *gdh* gene were used to carry out phylogenetic analysis together with reference sequences of representative isolates from GenBank (AB692779, Assemblage AI; JQ700433, Assemblage AII; AF069059, Assemblage BIII; JQ700432, Assemblage BIV; EF507637, Assemblage C; EF507628, Assemblage D; U47632, Assemblage E; AF069057, Assemblage F; AF069058, Assemblage G; and AF069060, *Giardia ardeae*). Analyses based on the neighbour-joining (NJ) and maximum-likelihood (ML) methods were implemented in MEGA 6 software [28]. The optimal base substitution model for *gdh* sequences of *Giardia*, estimated by MEGA 6, was TrN+G. Statistical support for nodes in the NJ and ML analyses were estimated by using 1,000 bootstrap replicates. Only bootstrap values higher than 50% were retained. Nucleotide composition and polymorphic sites were also analysed with MEGA 6. All sequences generated during this study were submitted to GenBank (Accession Numbers KJ741292—KJ741328).

### Statistical analysis

Statistical analyses were performed using R 3.0.2 and SAS 9.4. The Mantel–Haenszel Chi-square test was applied to check which variables were associated with positive results for *Giardia*. The significance level was set at 5%.

## Results

### Detection and typing of *G*. *duodenalis* in faeces

The prevalence of *G*. *duodenalis* by microscopy in the human samples was 8.9% (34/380). The *gdh* gene was successfully amplified and sequenced from the genomic DNA extracted from 27 samples (26 of the 34 samples positive by microscopy and one of the 34 negative samples). The *bg* gene was amplified in 16 (59.3%) of the 27 samples positive for the *gdh* gene. Typing of *G*. *duodenalis* identified one patient (3.7%) infected with assemblage AI, one (3.7%) with BIII, six (22.2%) with AII and 19 (70.4%) with BIV (Table 1).

**Table 1:** Detection of *G*. *duodenalis* in human faeces, dog faeces and vegetables using microscopy or the Merifluor Kit and assemblages found by PCR-RFLP and sequencing in Southern Brazil.

*Samples*	*Origin*	*Microscopy or Merifluor Kit*	PCR Detection/PCR-RFLP Β-giardin gdh*		Sequence*
E39	Human	+	−/NR	+/ND	A I
C42	Human	+	+/ND	+/B	B III
C54A	Human	+	+/A	+/A II	A II
C65	Human	+	+/A	+/A II	A II
C30	Human	+	+/A	+/A II	A II
F17	Human	+	+/A	+/ND	A II
E2	Human	+	+/A	+/A II	A II
I5	Human	+	−/NR	+/A II	A II
CF5-1	Human	+	+/B	+/B	B IV
E50	Human	+	+/B	+/B	B IV
E43B	Human	+	+/ND	+/B	B IV
C31A	Human	+	−/NR	+/ND	B IV
C10	Human	+	+/B	+/B	B IV
E74A	Human	+	+/B	+/B	B IV
E72A	Human	+	+/B	+/B	B IV
C56A	Human	+	+/B	+/B	B IV
F24	Human	+	−/NR	+/B	B IV
C63	Human	+	+/B	+/B	B IV
C69	Human	+	−/NR	+/B	B IV
C29	Human	+	−/NR	+/B	B IV
C54	Human	+	−/NR	+/B	B IV
C64	Human	+	+/B	+/B	B IV
E23	Human	+	−/NR	+/ND	B IV
C74B	Human	+	+/B	+/B	B IV
B36	Human	+	−/NR	+/ND	B IV
F30	Human	+	−/NR	+/B	B IV
F5	Human	−	−/NR	+/ND	B IV
F6	Human	+	−/NR	−/NR	NR
C47	Human	+	−/NR	−/NR	NR
C51	Human	+	−/NR	−/NR	NR
C25A	Human	+	−/NR	−/NR	NR
E9	Human	+	−/NR	−/NR	NR
B8A	Human	+	−/NR	−/NR	NR
B43	Human	+	−/NR	−/NR	NR
C44	Human	+	−/NR	−/NR	NR
DJ8	Dog	+	−/NR	+/ND	B IV
DJ5	Dog	+	−/NR	+/ND	C
DJ1	Dog	−	−/NR	+/ND	D
H11	Vegetable	−	−/NR	+/ND	B IV
H12	Vegetable	−	−/NR	+/ND	B IV

ND: Not detected NR: Not performed

* Comparison of the ability to differentiate into assemblages by PCR-RFLP and sequencing (*gdh* gene) using Z test with p = 0.0008.

Regarding the dog samples, 6.3% (2/32) showed positive results by microscopy and assemblages BIV and C, and in 3.2% (1/32) with negative microscopy was observed assemblage D (Table 1).

### Detection and typing of *G*. *duodenalis* in vegetables

**Fig 1:**
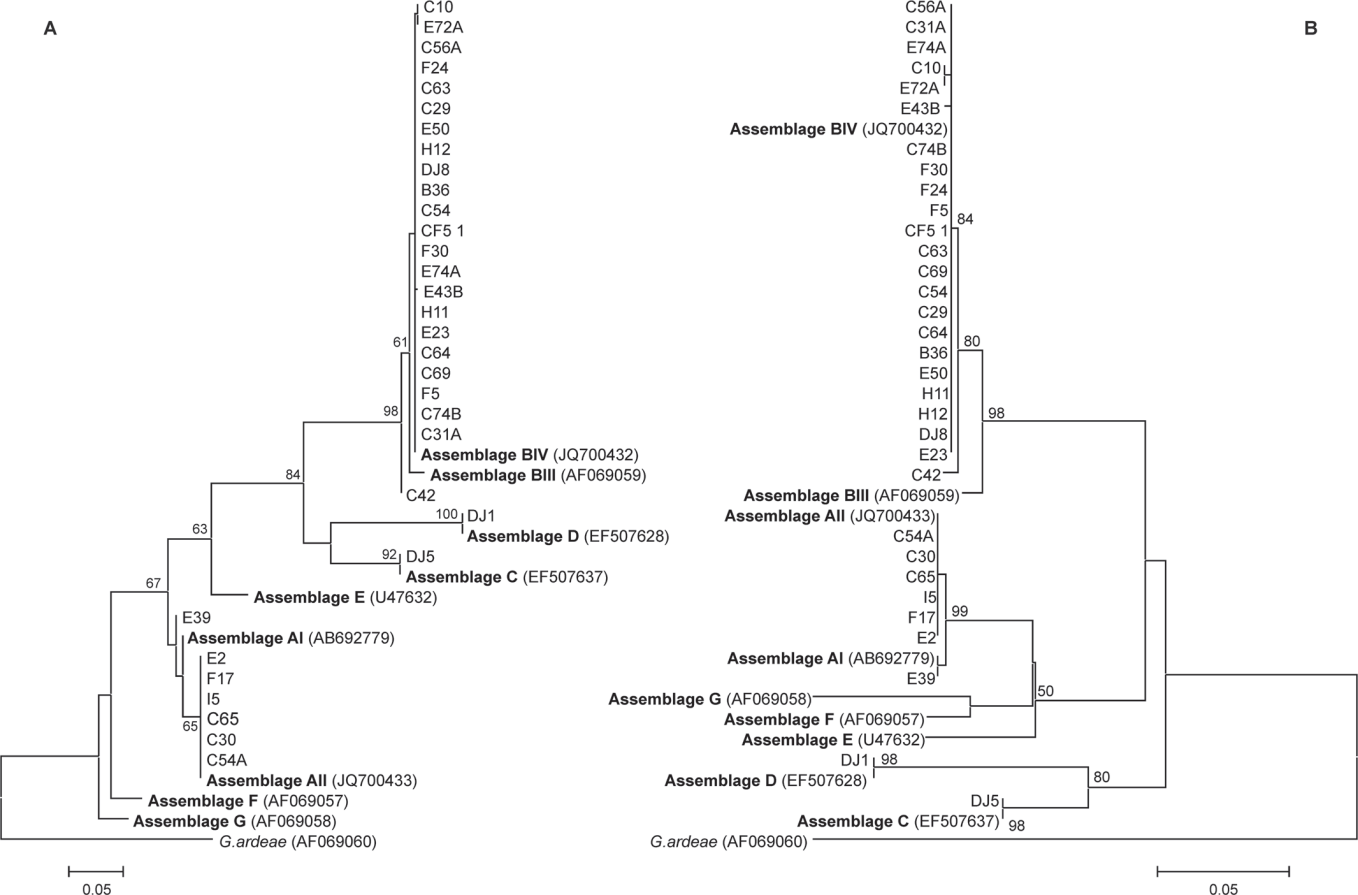
Dendrograms of *Giardia duodenalis* based on nucleotide sequences of the *gdh* gene. Trees were constructed using the neighbour-joining (A) and maximum-likelihood (B) methods, implemented with MEGA 6. Numbers above or below branches indicate bootstrap values (%), estimated from 1,000 resamplings of the sequence data; bootstrap values <50% are not shown. Taxon names indicate species (E, C, B or F = human; D = dog; H = vegetables), followed by identification numbers. Reference sequences are in bold.

**Fig 2:**
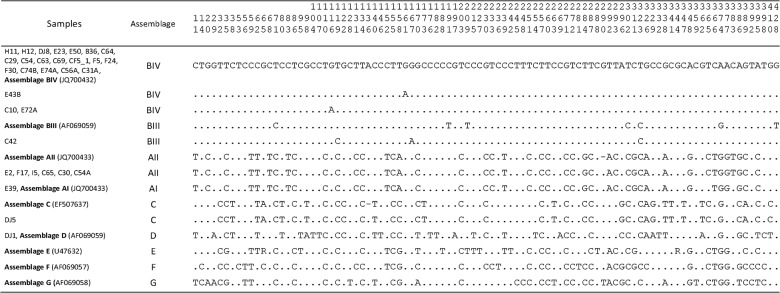
Polymorphism at 93 nucleotide sites (and two indels) in partial sequences of the *gdh* gene (431 bp) in samples of *Giardia duodenalis* isolated from human faeces, animals and vegetables, from southern Brazil. The entire sequences can be found at GenBank with the accession numbers KJ741292-KJ741328. Taxon names indicate species (E, C, B or F = human; D = dog; H = vegetables), followed by identification numbers. Reference sequences are in bold.

Two *Lactuca sativa* (2/11; 18.2%) samples were positive only by PCR. Assemblage BIV was identified in both samples by sequencing (Table 1, Figs. 1, 2).

### Immunologic, molecular and microbiologic analyses of water

All water samples showed negative results for *G*. *duodenalis*. Three samples (A1, A2, A3) presented total coliforms ranging from 400 to 2600 CFU/100 mL. In one sample (A1), the presence of *Escherichia coli* at a concentration of 100 CFU/100 mL was also observed. Samples A1 and A2 showed 139 and 93 mgPtCo/L colour, respectively, and turbidity under the limits allowed by CONAMA [22] (under 40 NTU).

In samples A4 to A44 the physical-chemical and microbiologic parameters were within the limits established by Ordinance 2914/2012 of the Ministry of Health for drinking water for human consumption [21].

### Relation between studied variables and positivity for *G*. *duodenalis*

Table 2 shows the analysis of the relationship between the prevalence of *G*. *duodenalis* and different variables studied. Age was the only variable associated with the presence of giardiasis (p = 0.00054), and was more frequent among children up to 12 years old.

**Table 2:** Analysis of the relationship between the prevalence of *G*. *duodenalis* and different variables using the Mantel-Haenszel chi-square test.

**Variables**			***Giardia***				**OR**	***p***
			**Negative**		**Positive**			
			**(n = 345 90.8%)**		**(n = 35 9.2%)**			
			**n**	**%**	**n**	**%**		
**Age**								
	5 months to 5 years old		63	16.6	16	4.2	7.07	0.00054*
	6 to 12 years old		95	25.0	12	3.2		
	13 to 17 years old	Ref	58	15.3	1	0.3		
	>17 years old		129	33.9	6	1.5		
**Gender**								
	Female		213	56.1	19	5.0	0.78	0.37382
	Male	Ref	132	34.7	16	4.2		
**Filtered water consumption**								
	No		205	53.9	22	61.1	1.07	0.99991
	Yes	Ref	140	36.8	13	38.9		
**Dog in household**								
	No		165	43.4	18	4.7	1.08	0.95447
	Yes	Ref	180	47.4	17	4.5		
**Cat in household**								
	No		289	76.1	32	8.4	2.14	0.31282
	Yes	Ref	56	14.7	3	0.8		
**Water cistern at home**								
	No		133	35.0	18	4.7	1.59	0.25341
	Yes	Ref	212	55.8	17	4.5		

OR: Odds Ratio;

*p< 0.05 (Mantel-Haenszel chi-square test).

Ref: variables that have the lowest prevalence were considered as reference.

Positive results were based on parasitological and molecular analyses.

### Comparative analysis between sequencing and PCR-RFLP results

The sequencing and PCR-RFLP results showed 100% concordance, but sequencing was significantly more sensitive (p = 0.0008) (Table 1). Seven (AI and AII) and five (AII) samples were identified by sequencing and PCR-RFLP, respectively. Most of the sequences analysed belonged to assemblage B (one BIII and 22 BIV by sequencing, and 15 B by PCR-RFLP). Two sequences originating from dogs were assigned to assemblages C and D by sequencing only (Table 1).

### Phylogenetic analysis and relationship among the different links in the epidemiological chain of *G*. *duodenalis*

Alignment of the 431 bp nucleotide sequence of the *gdh* gene showed 93 polymorphic sites, which was sufficient to discriminate between different assemblages (Fig. 2). As can be seen in this figure within each assemblage, samples revealed low levels of polymorphism. Analyses conducted by NJ and ML confirmed these results and showed six assemblages of *G*. *duodenalis* (AI, AII, BIII, BIV, C and D), among which BIV was a common genotype among different sources or hosts (humans, animals and vegetables), and the one most often found in humans. These samples were grouped in a single clade, with high bootstrap values, 61% for NJ and 84% for ML, indicating a relation between them (Fig. 1).

## Discussion

In Brazil there have been no studies reporting the relationship between different links in the epidemiological chain of *G*. *duodenalis*, in the same focus of endemicity, using molecular techniques. By *bg* and *gdh* PCR-RFLP and *gdh* sequencing it was possible to verify in an urban area of Southern Brazil the relationship between different links (dogs, humans and vegetables) in the transmission chain of *G*. *duodenalis*. The occurrence observed for this parasite was 9.2% for humans and 9.4% for dogs which is within the expected limits, since infection rates ranging from 0.9%–42.9% in humans and from 3.0–64.3% in animals were previously reported [2]. Of the three dogs that were positive for *Giardia*, one presented assemblage BIV, also found in humans and vegetables, showing a relationship between them. For this assemblage, the identical sequence associated with robust phylogenetic groups allows us to infer zoonotic potential of this parasite in the study area, though statistical analysis showed no association between dog/cat ownership and *G*. *duodenalis* prevalence. Other authors [29–32] also observed no statistical association between the frequency of giardiasis and the presence of dogs at home.

The finding that showed BIV to be the most frequent assemblage can be related to its greater spread. Kohli et al (2008) [13], in North-eastern Brazil, reported that children infected with assemblage B eliminate larger numbers of cysts compared to those infected with assemblage A. A higher frequency of assemblage B has also been observed by other authors [29,33,34]. In contrast, Volotão et al. (2007) [10], Souza et al. (2007) [35] in Brazil and Zhang et al. (2012) [36] in China found a predominance of genotype A, when specimens from humans and dogs were analysed. These data show geographical variations in the predominance of assemblages of *Giardia* that may be explained by the difference in habits and behaviours of individuals, prior exposure, and factors linked to genetics of the host and/or of the parasite [2,37].

One data (unpublished) from our laboratory that underscores the importance of habits, behaviours, exposure time in an area with a high risk of transmission, in the maintenance and circulation of assemblages is the fact that a patient of the study, at baseline and after the first treatment had showed AII assemblage and after second treatment BIV, with the BIV remaining after the third treatment. This individual was the owner of a dog that was not infected, but was responsible for the cultivation of vegetables and the owner of the residence where the well water showed a total coliforms and *Escherichia coli* index above those allowed by CONAMA [22].

The vegetables were irrigated by sprinkling, two to four times a day, indicating that during the process of irrigation and cultivation, the time and frequency of exposure may concentrate *Giardia* cysts originating from a source of contamination (water for irrigation) that probably had small numbers of cysts undetected by the performed analyses. Irrigation was performed with water (A1) originating from a shallow well, without adequate protection and basic cleaning care and localize at an ideal distance from, but at a lower level than the septic tanks, becoming vulnerable to anthropogenic contamination [38]. Although the water did not show *Giardia sp* cysts, the presence of total coliforms and *E*. *coli* index above those allowed by CONAMA [22] indicate contamination of the drinking water with faecal material [39]. Karon et al. (2011) [19] also associated cases of giardiasis with the use of water with the number of total coliforms and turbidity levels above the limit, but without *Giardia* cysts. The contamination of vegetables reported in the present work may be due to the characteristics of the well and by fact that the elimination of human faeces is mainly through septic tanks, and that animals roam freely around the peridomicile, contaminating the soil, and consequently, the well by surface run-off during rains. As the waters originating from shallow wells, springs and artesian wells showed good appearance, are translucent, tasteless and odourless, they provide consumers with a sense of security, as a result of which they may not treat or even disinfect water intended for consumption or for use in the irrigation of vegetables that are consumed raw [40].

The only variable associated with giardiasis was age, being more common in children up to 12 years old, which is consistent with findings of other authors [29,30,41]. For the other variables investigated (gender, filtered water consumption, presence of pets and has water cistern at home) there was no association with the frequency of giardiasis, as also observed by other authors [30–32].

This is the first study in Brazil that reports the connection among different links in the epidemiological chain (humans, dogs and vegetables) in the transmission dynamics of *G*. *duodenalis* in the same geographic area. The assemblage most frequently observed was BIV which was identical among the different links of the epidemiological chain.
